# Security Mechanisms of a Mobile Health Application for Promoting Physical Activity among Older Adults

**DOI:** 10.3390/s21217323

**Published:** 2021-11-03

**Authors:** David Bastos, José Ribeiro, Fernando Silva, Mário Rodrigues, Carlos Rabadão, Antonio Fernández-Caballero, João Paulo Barraca, Nelson Pacheco Rocha, António Pereira

**Affiliations:** 1 Computer Science and Communications Research Centre, Polytechnic Institute of Leiria, School of Technology and Management, 2411-901 Leiria, Portugal; david.bastos@ipleiria.pt (D.B.); jose.ribeiro@ipleiria.pt (J.R.); fernando.silva@ipleiria.pt (F.S.); carlos.rabadao@ipleiria.pt (C.R.); apereira@ipleiria.pt (A.P.); 2 Institute of Electronics and Telematics Engineering of Aveiro, Higher School of Technology and Management of Águeda, University of Aveiro, 3810-193 Aveiro, Portugal; mjfr@ua.pt; 3 Departamento de Sistemas Informáticos, Universidad de Castilla-La Mancha, 02071 Albacete, Spain; antonio.fdez@uclm.es; 4 Biomedical Research Networking Centre in Mental Health (CIBERSAM), 28029 Madrid, Spain; 5 Department of Electronics, Telecommunications and Informatics, Institute of Telecommunications, University of Aveiro, 3810-193 Aveiro, Portugal; jpbarraca@ua.pt; 6 Department of Medical Sciences, Institute of Electronics and Telematics Engineering of Aveiro, University of Aveiro, 3810-193 Aveiro, Portugal; 7 INOV INESC INOVAÇÃO, Institute of New Technologies—Leiria Office, Apartado 4163, 2411-901 Leiria, Portugal

**Keywords:** older adults, health, healthy lifestyle, smart city, data privacy, integrity, confidentiality, security mechanisms

## Abstract

Physical activity contributes to the maintenance of health conditions and functioning. However, the percentage of older adults who comply with the recommendations for physical activity levels is low when compared to the same percentages on younger groups. The SmartWalk system aims to encourage older adults to perform physical activity (i.e., walking in the city), which is monitored and adjusted by healthcare providers for best results. The study reported in this article focused on the implementation of SmartWalk security services to keep personal data safe during communications and while at rest, which were validated considering a comprehensive use case. The security framework offers various mechanisms, including an authentication system that was designed to complement the pairs of usernames and passwords with trusted execution environments and token-based features, authorization with different access levels, symmetric and asymmetric key cryptography, critical transactions review, and logging supported by blockchain technology. The resulting implementation contributes for a common understanding of the security features of trustful smart cities’ applications, which conforms with existing legislation and regulations.

## 1. Introduction

In the past decades, there has been an increment of the proportion of older adults that make up nations’ population in almost every country [1]. One of the reasons for this is the significant increase in life expectancy from the 1950s onward, with studies predicting that it will continue to increase, reaching the ninety-year mark by 2030 [2]. Consequently, older adults will be required to remain in the workforce for longer and to be independent and autonomous when they leave, since there will be fewer young people available to care for them. This problem is increased by the fact that life expectancy is not directly translated to functioning capacities, but even if deterioration of faculties (both physical and mental) is unavoidable, steps can be taken to soften it. Concepts such as ageing in place [3] or active ageing [4] consider that the maintenance of patterns of activities and values typical of middle age can optimize opportunities for social participation, health conditions, and the safety of the individuals as they age [4,5].

The use of smart technologies is increasingly considered an important mean to promote active ageing [6,7]. Therefore, many researchers aimed to develop new services based on information technologies (IT), such as ambient assisted living (AAL) [8], or smart cities’ applications to promote age-friendly cities and communities [9] by facilitating the mobility of older adults [10,11,12,13,14,15,16] or by promoting healthy lifestyles (i.e., physical activity) [17,18].

The main goal of the SmartWalk project [19] is precisely to use the smart cities’ infrastructures to encourage older adults to increase physical activity, as it is related to improved health conditions and functioning [20,21,22], safely guided by healthcare providers. For that purpose, SmartWalk provides a selection of routes for the users to walk on. During a walk, various types of data are collected by the users’ smartphones and the sensors connected to them [23], which are stored and made available for consultation by healthcare providers to prepare personalized recommendations. Systems aiming to constantly monitor individuals’ activities such as the SmartWalk have the potential of putting the privacy of their users at risk, since personal data are transferred through the internet [24]. Therefore, secure data transmission together with the guarantee that the stored data should only be accessed by people who are authorized are important requirements. If these requirements are not satisfied, information such as the location of a person at a given time might be used for nefarious purposes, from phishing or hustling schemes to knowing when and for how long the individuals are out of their homes.

In this context, the study reported by this article implemented and validated a security framework for the SmartWalk system, which was informed by two research objectives: to verify the feasibility of implementing security mechanisms using current technologies to support applications targeting the mobility, monitoring, and healthy lifestyles of older adults in the context of smart cities and to identify the required security features not only to guarantee conformance with existing legislation and regulations but also to increase the trust of potential end users.

The main contribution of the reported study consists of a set of security mechanisms, including authentication mechanisms that, in addition to the username and password pair, integrate trusted execution environments and token-based authentication features, flexible authorization mechanisms to provide secure data access to authorized entities with different access levels, symmetric and asymmetric key cryptography to protect data, trustful mechanisms to review the critical transactions, and logging mechanisms supported by blockchain technology. Moreover, the study also contributed for a common understanding of the required security features to guarantee that trustful smart cities’ applications targeting older adults conformed with existing legislation and regulations.

The next section briefly reviews the current state of the art and how it influenced the research work reported by this article. Afterwards, Section 3 overviews the SmartWalk system and presents the methods of the reported study. Section 4 focuses on the implementation of the security mechanisms, including their rationales and how they work, as well as their validation. Finally, Section 5 presents a discussion of the results and draws a conclusion.

## 2. Background and Related Work

Due to their capabilities, in terms of both data processing and data acquisition, namely, by built-in sensors, as well as the relative easiness to connect wirelessly external sensors, smartphones are useful tools for healthcare provision [25]. Particularly, considering their increasing omnipresence related to individuals’ tendency to always keep their smartphones with them, smartphones are adequate to monitor parameters and patterns related to health conditions, without the need for intrusive devices. These features allow smartphones to be used to collect extensive data from users who already own a smartphone and who are accustomed to using it.

Older adults might use applications geared towards the general population and reap their benefits, but, considering their age and health conditions, they have additional requirements that need to be satisfied. Consequently, specific applications are required, such as Medicine Alert [26], which helps the users to better manage their medication (e.g., when to take a medication or when to refill a prescription); Blood Pressure Companion [27], which allows users to enter blood pressure measurements and easily visualize and analyze their blood pressure history; or the Dip.io application [28], which uses a smartphone camera and a special kit to allow users to perform urinalyses in the comfort of their own home, with the results being sent to a cloud server for classification.

The security dimension must be considered, not only to conform to the General Data Protection Regulation (GDPR) [29] but also because the impact of improper data handling might be very high, including public exposure, scrutiny, and maybe shame for the users. The sole fact that data may eventually be leaked or accessed by others is a detrimental aspect that creates resistance in the adoption of any data-gathering system [30,31]. Recently, this was observed in the applications implementing the decentralized privacy-preserving proximity-tracing (DP-3T) protocol [32]. While the protocol only results in the broadcast of anonymized beacons, without providing the direct possibility of correlating beacons to an individual, because of other applications that may not be so careful [33], or the work of security researchers that explore specific situations that are not trivial to reproduce, citizens may be highly suspicious of using tracking devices. Interestingly, telecom operators and several third parties have managed to keep their image clean, while gathering massive amounts of personal data [34].

Most importantly in a user’s mind is the fact that a mobile device is an object that can be stolen or lost. However, security threats are also related to malicious attacks (e.g., phishing attacks [35], ransomware [36], or insider attacks that might occur when an element of the organization uses their inside access for malicious purposes [37]), which require key measures to safeguard data security including the use of cryptographic methods, right in the design of the solutions, making it more difficult for an attacker to obtain personal data. State-of-the-art solutions employ encryption in all communication channels and data repositories [38,39] and allow access only after strong authentication and authorization with strict policies [40]. Moreover, trusted execution environments (TEE) prevent physical and software attacks performed on the main memory of the systems to explore backdoor security flaws [41].

Data sharing is important to optimize the services delivered to the citizens but may lead to security problems and the disclosure of privacy-sensitive information [42]. To face these challenges, there is the need of trusted thirds parties (TTP) [43] (e.g., a notary, who is trusted by a regulatory body to authenticate documents and signatures), as shown in the proposed file remotely keyed encryption and data protection (FREDP) [44], which uses private clouds as trusted parties or in the proposed multi-authority-based file hierarchy [45], which uses multiple trusted parties to improve security. As an alternative, blockchain technology allows secure decentralized storage and data management by deploying distributed ledgers among all parties to store records without third-party authorities [46,47] and has been the object of significant research in different application domains [48,49].

Moreover, data sharing also requires access control policies to restrict the access to specific information items according to the identification of the requesting entity, the categorization applied to the information, and other aspects including environmental or contextual attributes [50]. The policies are used to create effective access mechanisms, typically based on attribute-based access control (ABAC) [51] or role-based access control (RBAC) [52] models, and can be expressed by the extensible access control markup language (XACML) [53].

Cybersecurity is a constant arms race between attackers and defenders, and the defenders are at a disadvantage as they can only react when a new threat is unveiled. Therefore, despite all precautions and safety measures implemented in a system, it is still possible for an attacker using new methods to cause great damage before being detected and stopped. To lessen this gap, systems have been proposed that make use of neural networks that can use data from known types of attacks to learn and then use that knowledge to analyze network traffic and detect if an attack is occurring or not, even if it is through a method not seen before [54].

Despite the importance of data privacy, integrity, and confidentiality, the smart city developments aiming to promote the mobility and physical activity of older adults do not conveniently address these questions [24], as can be observed in various studies [10,11,12,13,14,15,16,17,18]. Therefore, the present study aimed to contribute with solutions to surpass this gap.

## 3. Materials and Methods

Secure data transmission and the guarantee that the stored data should only be accessed by people who are authorized are important requirements of the SmartWalk system. Therefore, this study took inspiration from some of the current approaches reported in the previous section to guarantee the privacy, integrity, and confidentiality of the personal data of the SmartWalk users.

### 3.1. The SmartWalk System

**Figure 1:**
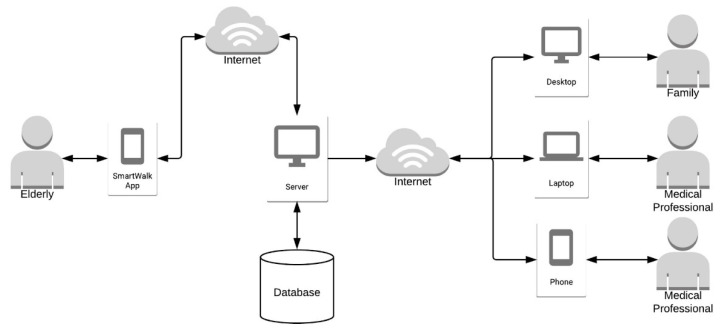
SmartWalk architecture.

The architecture of the SmartWalk system [19,55,56] exploits the client–server and microservice models over a cloud infrastructure (Figure 1). An important component of the SmartWalk system is a mobile application, the SmartWalk app, which aims to encourage older adults to perform physical activity (i.e., walking in the city) while under the remote supervision of healthcare providers. The mobile application makes use of the sensors already presented in a typical smartphone and connects external sensors of a body area network (BAN) (whose implementation details were presented in [19,23]).

In terms of communications, the architecture was designed to make use of the infrastructure provided by a smart city, so that the collected data can be easily transmitted without relevant communication costs. The data collected by the SmartWalk app are sent to the SmartWalk server infrastructure, which is responsible for their processing and storage. Moreover, the SmartWalk server infrastructure also provides a back-office web application designed for healthcare providers (e.g., physiotherapists), so that they can analyze care receivers’ information (e.g., health conditions, relevant events gathered by the sensors of the SmartWalk app, or routes that were performed) or to plan fitness regimens by scheduling walks with a diversity of routes.

In terms of implementation, the SmartWalk app was designed to be installed on Android smartphones. The earliest version of Android compatible with the SmartWalk app is version 5.0 (application programming interface—API—level 21). The SmartWalk app provides a single interface for older adults, through which they interact with healthcare providers, receive routes, and send tracking data at selected instants. The routes, which can be created using the My Maps service from Google or the geojson.io website, are downloaded as keyhole markup language (KML) files and made available as tracks for hiking or small walks.

Google’s awareness API [57], supported by global positioning system (GPS) or triangulation based on Wi-Fi networks, was used to register whether the users were moving or not and, when moving, whether their movement conformed to the planned routes. In turn, the Google Fit API, by using the accelerometer sensors that all modern smartphones have, records the different types of activity during the walk (i.e., walking, running, or resting). Moreover, the Snapshot API provides the current context when needed (e.g., to check for the users’ current locations, whether they are running or whether their headphones are plugged in). Moreover, the Fences API was used to implement geofences, by which a geographical area is defined, and the application receives a callback when the smartphone enters or exits that area, which can be expanded by enabling the creation of fences from other context types. It also allows for different fences to be combined using Boolean operators. Finally, Bluetooth low energy (BLE) was used to connect a BAN that includes a heart rate/pulse oximeter sensor that registers both the number of beats of the heart per minute and the level of oxygen saturation in a persons’ blood [23].

In the design of the interface of the SmartWalk app attention was paid to assure that the graphical elements (e.g., the route’s map) are large enough and are sufficiently separated from one another to facilitate the interaction [58]. The screens cannot be rotated (to landscape mode) since changing the screen mode can be confusing, especially if it is done without the user noticing [59]. Some screens also do not turn off by themselves for the same reasons. Moreover, the back button is always available for use and serves as a fallback mechanism when the users do not know how to handle something. It allows the users to go back to the main screen, which serves as the safe point of the application, where all the available features are presented [60]. Alongside the visual medium, the auditory medium also deserved special attention. The application makes use of auditory cues for various purposes, and those cues were chosen with care, so that the users can correctly associate sounds to actions [61]. For example, actions that require immediate attention from the users are associated to an audio cue that reinforces the urgency, and thus the same audio cue is not used for less important tasks. Additionally, care was taken to the time required to use audio cues correctly and to guarantee that audio cues are not used too much (or the users may just turn off the sound in annoyance) nor that they are seldom used (in which case the users would ignore them).

The SmartWalk server infrastructure is a platform with diverse software components, including the SmartWak server and the back-office application, hosted in an infrastructure provider or at local premises, which stores all the data collected by the SmartWalk app. These data are stored in a structured query language (SQL) database, which conforms to the Fast Healthcare Interoperability Resources (FHIR) [62], a healthcare interoperability standard to guarantee the integration of disparate healthcare systems and the interchange of clinical information between different healthcare providers and organizations. The SmartWalk server also provides the services needed by the SmartWalk app (e.g., provision of the KML files containing the routes) through RESTful APIs. These RESTful APIs also support a back-office application to be used both by healthcare providers and care receivers. For the healthcare providers, the back-office application allows the creation of routes (e.g., using the My Maps service from Google and then uploading the generated KML files), the planning of a fitness regimen by scheduling walks on various routes, and the visualization of care receivers’ information, such as health conditions, routes they have been walking or not walking, and an historical graph on the pain levels of the various body parts of the care receivers. In turn, the care receivers might access their own information and decide what can be shared with their formal healthcare providers or relatives.

### 3.2. Security Mechanisms Implementation and Validation

Any application that handles sensitive data must find ways to guarantee the respective privacy, integrity, and confidentiality, and it is in fact legally obligated to do so in many jurisdictions [63]. In particular, the disclosure of private clinical information is a risk that must be considered. Even though it is impossible to create a one-hundred-percent secure system, security threats can be mitigated by implementing a range of security procedures.

Following the applicable legislation and regulations (e.g., GDPR) with regard to the processing of personal data (e.g., movement data), the study reported by this article was focused on security mechanisms to keep personal data safe during communications and while at rest, allowing only authorized entities access to the information resources and preventing illegal access attempts and interferences. This means that each data operation must be properly authenticated, using an adequate method for that purpose. Moreover, when required, the users themselves could authorize the access of subsets of personal health information to specific healthcare providers (e.g., their general practitioners), which implied the implementation of flexible mechanisms to provide secure data access to authorized entities, with different access levels. After the definition of the SmartWalk security requirements, the authors identified current technologies that could help accomplish those requirements. As a rationale, it was assumed that it would be beneficial to make use of existing solutions that have been tested, put into use, and found to be reliable and safe, instead of creating new ones.

The authentication based on a username and password pair must be complemented with additional mechanisms to increase robustness. In this respect, the Google attestation service and the TEE available in Android devices were used to reinforce the username and password authentication. In addition, a token-based authentication mechanism was also implemented, in which the username and password authentication are used to obtain a token (i.e., a JSON web token (JWT)), allowing access to specific resources without introducing the pair of a username and a password for a certain period.

Data protection during communications and while at rest is assured using cryptographic techniques, including symmetric key cryptography such as data encryption standard (DES), advanced encryption standard (AES), and asymmetric key or public key cryptography (e.g., the X.509). Given that it is easy to create fraudulent digital content, cryptography methods were complemented with TTP aiming to review all critical transaction communications in terms of identity authentication. Trustful relationships also require the validation of the log history to verify its integrity. In this respect, secure logging features that enable the creation of an activity trail for multiple entities using blockchain technology were implemented.

To validate the proposed implementation, a comprehensive use case that requires the installation of the SmartWalk app and the transmission of personal data to the SmartWalk server was used to verify the security features of the current implementation. Moreover, to study the impact of the security mechanisms on the usability of the SmartWalk app, the Apache JMeter version 5.4.1 program was used to simulate one hundred users communicating with the server in a short period of time. The simulations were designed to determine the connection (i.e., the time taken to establish a connection with the SmartWalk server) and latency (i.e., the time from the moment a request is sent to the moment the respective response is received) metrics, being the users represented by a single thread each, running concurrently with all the others, with each thread sending a JSON file with simulated health data with a size of 7.5 megabytes.

## 4. Results

### 4.1. Implementation of Security Mechanisms

The first barrier to consider is the widespread authentication mechanism based on the use of a pair of a username and a password. When the users enter the main screen of the SmartWalk app, they must login. Permissions are asked only whenever they are necessary and with a clear explanation of their purpose, so that they are transparent to the users, enabling them to make informed decisions. Full GPDR compliance is observed, allowing users to access data in an open format. Furthermore, data are deleted when the users stop interacting with the system or when they request deletion. An additional step we aimed to consider was the addition of consent receipts when data enrolls. We wished to align this feature with the work developed at the Kantara Initiative adapted to our scenario. These receipts can play an important part in the transparency and in clarifying the purposes associated with the data collected, in addition to represent the allowance to discretely collect data as mandated by current European laws. An external TTP can be used as neutral party to store the receipts.

To ensure the legitimacy of the data being transmitted, the SmartWalk app makes use of the Google attestation service and the hardware TEE. For older devices, limited features are available through libsodium. The SmartWalk app generates an asymmetric key, which remains present until the application is uninstalled, and upon start it provides both the public key, a signature over a Nonce, and the result of the attestation to the SmartWalk server. The asymmetric key is used to sign data, ensuring their origin by cryptographic means.

Similar mechanisms are presented in the Back Office Application. In this respect, in addition to the username and password, pairs of asymmetric keys will be used by the SmartWalk Server and Back Office Application to allow selective access of data blocks. A new key pair may be generated whenever required, and the new key pair will supersede the existing ones for a given context. Each key is associated with a set of data elements and grants a specific entity with the permission to access these data elements.

Data related to the SmartWalk app and the back-office application are encrypted using a data encryption key (DEK) together with the AES256 in Galois/counter mode (GCM). These keys will rotate periodically with a configurable interval. The applications store the keys locally and upload them to a TTP, in a vault. This vault is encrypted according to the advanced encryption standard (AES) using a random key encryption key (KEK), which is then encrypted using the user’s private keys. The purpose is to store keys securely, allowing the use of multiple smartphones by the same user, or dealing with the loss of a smartphone. It is also in line with the recommendations from the Federal Information Processing Standards (FIPS) or the Health Insurance Portability and Accountability Act (HIPAA), as data are generated in a secure and authenticated manner and stored in an encrypted format.

The connections between the SmartWalk Server and the applications are provided by a transport layer security (TLS) connection, through which hypertext transfer protocol (HTTP) data are transferred. The authentication of the connections is achieved by using a challenge-based response approach with password-based key derivation function 2 (PBKDF2), or the asymmetric keys stored in the TEE.

**Figure 2:**
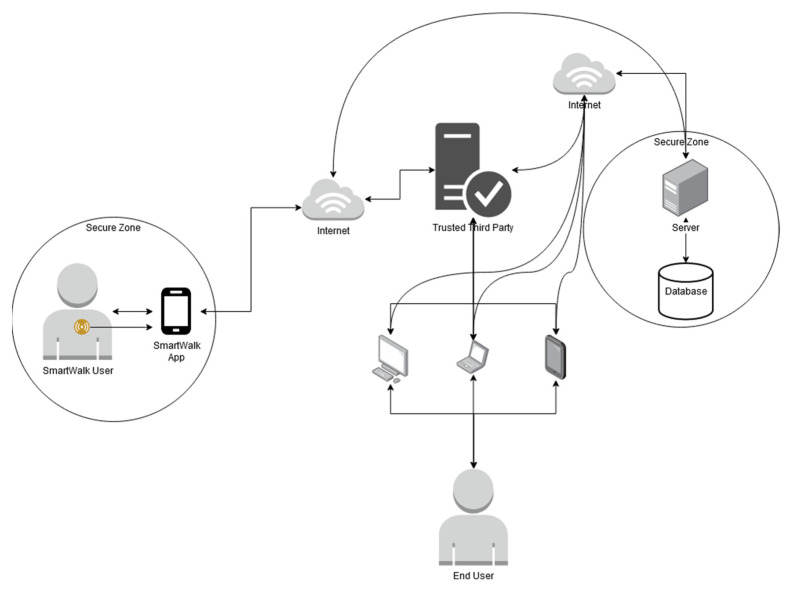
Security mechanisms architecture.

During any transaction between unknown parties, there is always a certain degree of distrust between them. One of the means to overcome this distrust is to have a third party that is trusted by all the parties to carry out the transaction (Figure 2). Conceptually, a TTP is a component that does not need to be managed by the entities that store the data and acts as a vault for keys. In terms of the implementation of the SmartWalk system, the TTP does not know the identity of the users or the data being exchanged while providing its function as a data gatekeeper. Furthermore, it is not necessary for a TTP to have the personal trust of the other parties, but it must be guaranteed, be it by reputation, legal, or other means, that it is trustworthy and will perform its assigned task honestly and without taking advantage of the trust given to it.

Since both the SmartWalk server and applications have a key pair and provide their public keys to the TTP to be certified, data can be sent from the applications to the server and vice-versa privately and securely using the TTP as intermediary.

Under this assumption, each user in the system is identified by a unique value, a universally unique identifier (UUID). A second set of UUIDs, generated periodically, is stored and provides data anonymization. Public keys, together with unique identifiers, are uploaded to the TTP. The certification using X.509 certificates is not required as users can easily validate the possession of the cryptographic material by comparing the key digests. However, a X.509 public key infrastructure might also be included in a future deployment, with advantages related to data sharing and online revocation.

Care receivers may choose to share their data with someone else (e.g., a healthcare provider or a relative). In some cases, it will not be the care receivers themselves but their informal care providers who define which information to share and with whom. However, in these cases, the informal caregivers can give information access to formal healthcare providers but are able to access the information.

Disclosing data is achieved by encrypting the DEK with the public key of destination user and publishing it to the TTP. A DEK can be shared as many times as required and will allow access to the data that it encrypts. When users (e.g., healthcare providers or care receivers) wish to access the shared data, they must authenticate themselves in the TTP vault by using their private keys and then obtain the respective DEKs. Given the infrastructure in Portugal, where all citizens have a smartcard with a strong authenticate mechanisms and all healthcare practitioners must use a personal smartcard to issue clinical acts (a standard practice mandated by law), the use of asymmetric cryptographic is a reality.

**Figure 3:**
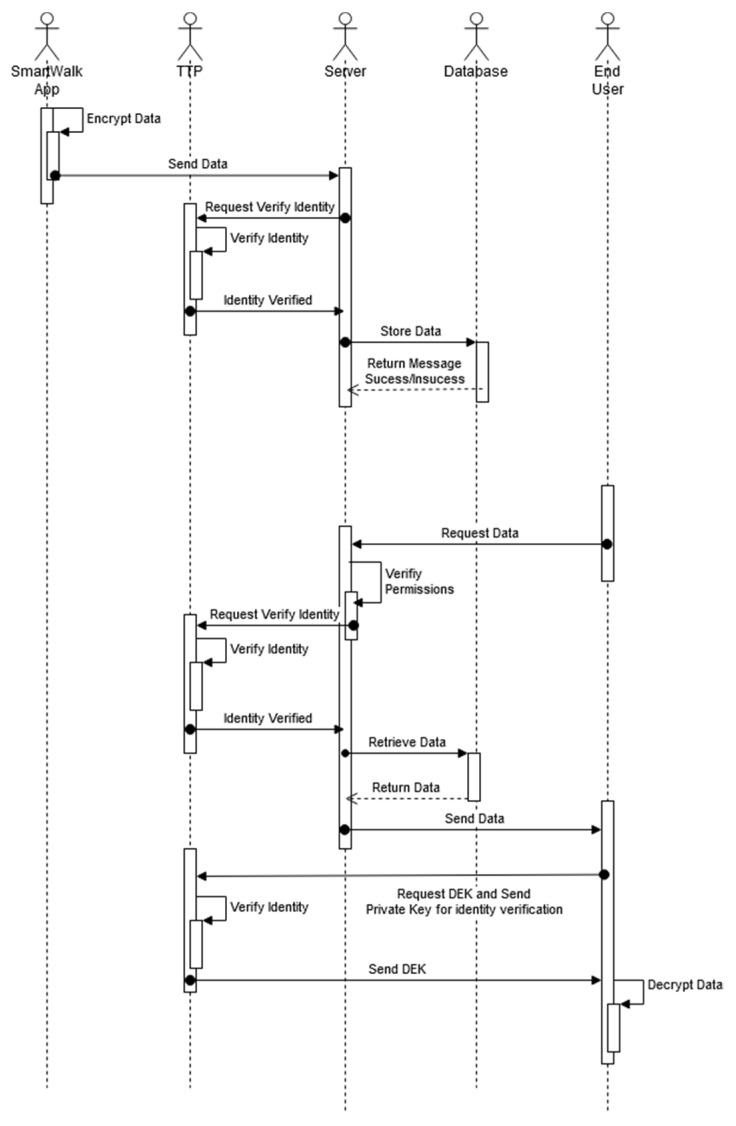
Data transmission diagram.

Figure 3 presents the data communication diagram of both the SmartWalk app and the back-office application. The data collected on the SmartWalk server are processed to comply with FHIR, and then they are securely stored. The access to the stored data implies the existence of a valid JWT that follows the standard RFC 7519 [64]. The veracity and validity of these tokens are verified by the communication parties using an encrypted elliptic curve private key. This avoids the need to store security information in the client side and guarantees that the requests are legitimate and recent.

Based on previous work [65], and in alignment with the FHIR requirements, all data are broken down into data objects with a very fine granularity. Each object complies to a class and is strictly identified, together with the schema and relation with other data objects. While it is possible to create data graphs, most data objects are hierarchical, with broader objects being composed by smaller objects representing the individual data fields. The advantage of this structure, and the extensive identification of all objects, is the capability to enforce policies that strictly restrict data access and manipulation. Following FHIR, all actions are strictly controlled by clear policies that are kept in a versioned repository and represented in XACML. A policy in XACML is based on actions, such as the route that was followed by a particular individual and the biometric data captured during the walk. Access is strictly restricted in accordance with existing relations, where only the delegated healthcare provider may access the data of a specific individual. The specificity provided by XACML and the fine granularity of the data model allow restricting access to specific objects, using role-based policies and attributed-based policies.

A useful example is the case in which healthcare providers of the same team may access some comments written by the assigned healthcare provider or the fitness plan of a given care receiver but will not have access to specific comments or the actual sensor data. At the same time, assigned family members can have selective access to some data so that they can help with the execution of the fitness plan. The creation of accesses is done by issuing fine-grained policies granting time-limited access to a specific item. This strongly limits the access to data by third parties.

Finally, all data accesses can be verified and audited at any point. For that, a previous work in which services use secure and authenticated connections to a log server [66] was exploited. The messages have integrity controls and are linked in hash chains following a strategy like a block chain (i.e., direct manipulations of the logs are easily detected as each block of log data has digests, and the blocks form a chain, by including the digests of the previous block). XACML policies in a versioned repository together with a secure log allow the validation of the actions done in the past. The developed system also supports the existence of additional TTPs for external log storage with different levels of privacy.

### 4.2. Security Mechanisms Validation

As in most security systems, the biggest risk presented in the described security implementation involves the people that use the system. People can cause major security breaches, be it by malicious intent or not. The users of the SmartWalk app can have their mobile device lost or stolen, and if the device is not locked up behind user authentication, any stored data in it is susceptible to be extracted by an outside party.

This is mitigated by the fact that the SmartWalk app only stores data locally temporarily if it is unable to connect to the SmartWalk server. Moreover, the use of current smartphones enforces the storage to be encrypted, and authentication is required to access the device. It was assumed that attacks to the TEE are possible, as demonstrated by previous works [67]. However, the cost of such attacks vastly surpasses the benefits of obtaining the collected data, and they will require a substantial level of sophistication.

Direct attacks to the SmartWalk server are also possible. The SmartWalk and accompanying database are in a restricted location where only trusted individuals with authorization should be able to access it, either physically or remotely. This is extremely important as the SmartWalk server contains the entirety of the data collected from the various users, as well as their identifications. However, attacks to the database would not be very helpful as all data regarding the users are encrypted with temporal keys. Relational information and metadata are only encrypted with standard keys by the SQL server, but this only signifies a small threat for the users. Moreover, data is associated with short-lived identifiers, and a different database is used to provide the relation between a UUID and a user. Accessing a table with location data will not allow disclosing personal identifiable information.

**Figure 4:**
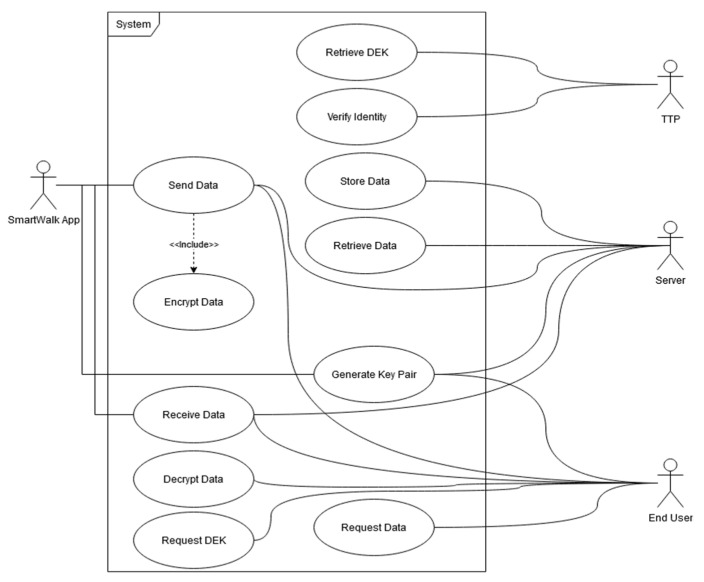
Security use case.

Considering the standard use of the SmartWalk app, Figure 4 presents a comprehensive use case that requires the installation of the SmartWalk app and the transmission of personal data to the SmartWalk server. After the user installs the SmartWalk app and starts using it, the application will generate a new key pair and a DEK and will share the DEK with the TTP. The TTP will create a random KEK, which will be encrypted with the user’s private key. When the application needs to send data to the SmartWalk server (e.g., after a walk is finished), it will send the data encrypted with this DEK and signed with the user’s private key. This guarantees that only authorized entities will be able to decrypt the data and that all the received data have guarantee provenances.

Although data are encrypted, while a user has a session open (especially a practitioner), several DEKs will be present in memory and may be potentially exploited. However, this would require a strong corruption of the program flow, potentially with attacks such as remote code execution (RCE). Most common attacks (e.g., SQL injections) would bring few benefits as data obtained would be encrypted, and although they could be used to destroy the data, this brings the attackers little benefit and can be easily countered by regularly backing up the database.

The TTP presents a major issue as it requires the DEKs to decrypt the data. However, this component was designed to guarantee that the encrypted data and the keys required to decrypt them are not in the hands of only one source. Thus, if an entity needs to make use of the data stored, it must always request authorization to obtain and use the keys. These limit the access of an unauthorized entity without supervision or accountability. If the TTP behaves correctly, it will require authentication using the smartphone application, which is backed by keys in the TEE or by the Portuguese Citizen Card, which is thought to be a secure platform. In environments without this infrastructure (e.g., outside Europe), due to lack of widespread hardware tokens in use, the authentication process would need to be carefully designed. Moreover, “man in the middle” attacks are not practical, firstly due to the use of HTTPS but also because keys, data, and authentication secrets are never transmitted in clear text. Even the process of allowing a user access to personal data only results in the submission of a DEK signed by the destination user’s public key.

Additionally, using a token-based method reduces the burden and insecurity of repeatedly submitting the users’ credentials. Tokens are only valid for a limited (short) time, which means that replay attacks can be avoided (in certain scenarios). Furthermore, these tokens are both revocable and refreshable. Therefore, it is believed that token-based authentication has the potential to provide tighter security.

The collected data are stored in a database of the SmartWalk server infrastructure, and users with authorization have access to the encrypted data but still need access to a DEK for the decryption. The SmartWalk server generates all the key pairs required for itself and will verify the end user’s private keys against their login information, which is required by their own authentication system. The access to the data stored in the SmartWalk server by any user requires the existence of an asymmetric key pair. Private keys, along with authentication of the SmartWalk server itself, are used to verify the users’ identity, which allows the access to the DEK stored on the TTP. This DEK is needed to decrypt any data requested.

**Figure 5:**
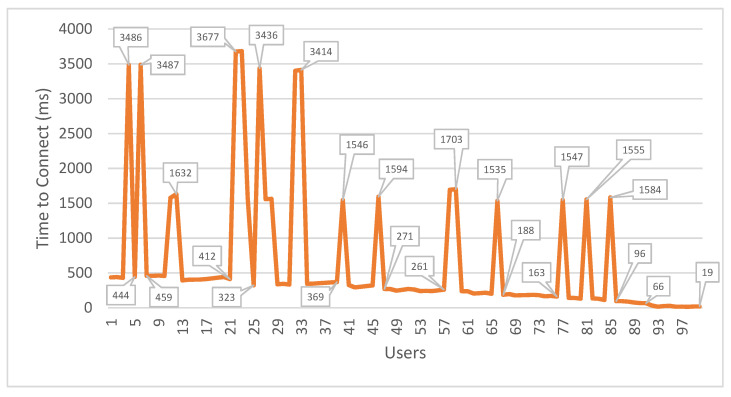
Connection time versus number of users.

**Figure 6:**
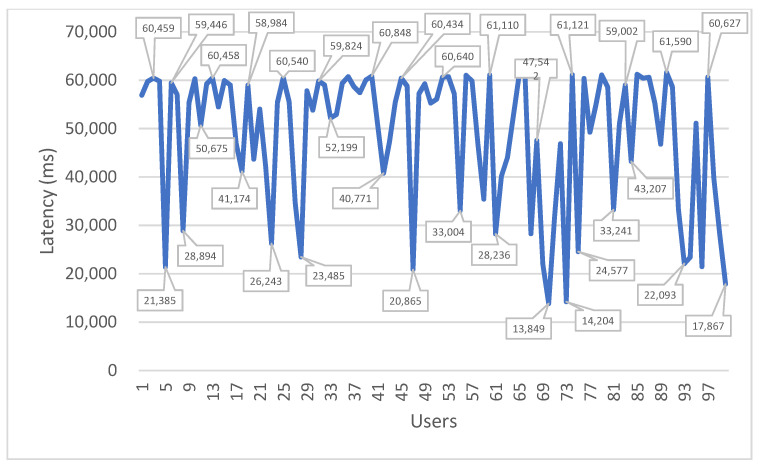
Latency time versus number of users.

All the mechanisms for data encryption and decryption as well as the verification of identities impact the SmartWalk performance, which is unavoidable; security requirements should nevertheless be implemented both for ethical and legal reasons. To verify how the mechanisms affect the usability of the SmartWalk app, simulations were performed using the Apache JMeter version 5.4.1 program to determine connection and latency metrics when one hundred users communicate with the server in a short period of time. The simulations were performed in a computer with a 1.9 GHz Intel Core i7-3517U processor and 8 GB of RAM, and they took 62 seconds from start to finish. The results are presented in Table 1, Figure 5 (connection metrics), and Figure 6 (latency metrics).

As can be observed, when there are many requests at the same time, the performance suffers; there are, however, ameliorating factors. First, there are situations where the users do not need to wait for a response from the server, and, as such, the inconveniences to the users are minimal. Secondly, data transmissions do not affect the usability of the application, as they can be performed even when the users are not actively using the application and are integrated in processes not observed to the user. Thirdly, this simulation was performed in a development environment, whereas the final deployment is more robust, and, therefore, a decrease in these values is expected.

## 5. Discussion and Conclusions

The SmartWalk was designed to make use of the infrastructures provided by a smart city and to take advantage of the advanced communication networks, namely, in terms of availability at reduced costs. The study reported by this article implemented a security framework for the SmartWalk system. Its first research motivation was investigating the feasibility of implementing security mechanisms using current technologies to support applications targeting the mobility, monitoring, and healthy lifestyles of older adults in the context of smart cities.

Given that smart cities’ infrastructures are public and easily accessible, it is important to consider robust security mechanisms to guarantee the privacy, integrity, and confidentiality of personal data. Even though many research studies identify privacy as a significant issue in smart cities, there is evidence [24] that the issues arising from the use of personal mobile and wearable sensors by applications addressing the mobility of older adults in smart cities (e.g., [10,11,12,13,14,15,16]), and for promoting healthy lifestyles in particular [17,18], are under-researched. Additionally, the privacy issues related to the gap among the digital competences of older adults and the privacy management requirements of mobile applications and related problems have not been conveniently solved yet [16].

A study of the best security practices was performed, and several mechanisms were implemented to secure the data generated by the SmartWalk app during communications and at rest, which are in line with solutions reported in the literature aiming to protect personal data of other types of smart city applications [68,69,70]. Moreover, an analysis of the implemented security mechanism was performed, and it was possible to conclude that the usage of different security technologies provides a framework that mitigates security threats and guarantees the privacy, integrity, and confidentiality of personal data. In terms of performance, the implemented security mechanisms do not affect the usability of the SmartWalk app since data transmission can be preferably done when the users are not actively using the application.

The proposed framework offers various security services: authentication mechanisms that, in addition to the pair of a username and a password, integrate trusted execution environments and token-based authentication features; flexible authorization mechanisms supported by XACML to provide secure data access to authorized entities with different access levels, being the fine granularity of specific objects defined by role-based policies and attributed-based policies; symmetric and asymmetric key cryptography to protect data during communications and while resting; trustful mechanisms to review all the critical transactions in terms of identity identification; and logging mechanisms using blockchain technology to provide data activity trails of all involved entities.

Developing smart city applications is quite complex and their requirements needed to be comprehensively systematized. Therefore, considering the second research motivation of the study reported by this article (i.e., to contribute for a common understanding of the security features of trustful smart cities’ applications that conformed with existing legislation and regulation), it is possible to conclude that several security requirements were identified and satisfied, including authentication, a flexible authorization mechanisms, data encryption, trustfulness, logging, and auditing. These requirements assure the preservation of identity, as well as of the location and queries of the citizens using smart-city applications. Smart cities present a huge potential to facilitate the mobility of older adults and promote healthy lifestyles. However, such potential might only be achieved if there are trustful applications, such as the one provided by SmartWalk, that do not put their users at risk.

A limitation of this study is related to the fact that a formal security analysis using an automatic tool (e.g., ProvVerif) was not performed to complement the validation of the security framework using a comprehensive use case. Another limitation of this study is the lack of evaluation in a real-life scenario. The SmartWalk system was developed in cooperation with the local authorities of Águeda, a municipality in the center of Portugal with around 50 thousand inhabitants, which is part of a region where the elderly population is increasing. The aim was to add SmartWalk to the existing services of the Águeda Smart City. Even though the implementation of the different components of the SmartWalk system was concluded, a real-world pilot study involving older adults was delayed due to the COVID-19 pandemic. However, a pilot study is planned for in the near future, which will measure the impact of SmartWalk, within the smart city paradigm, on the sedentary lifestyle of older adults.

## Figures and Tables

**Table 1:** Connection and latency metrics.

Metrics	Minimum (ms)	Maximum (ms)	Average (ms)
Connection	14	3683	650
Latency	13,849	61,590	48,872

## Data Availability

No new data were created or analyzed in this study. Data sharing is not applicable to this article.
